# Ferroptosis in vascular cognitive impairment: pathological mechanisms and traditional Chinese medicine interventions

**DOI:** 10.3389/fneur.2026.1862317

**Published:** 2026-09-25

**Authors:** Shiwen Zheng, Xiaobo Ji, Shicheng Zheng

**Affiliations:** 1 Department of Geriatric Cerebrovascular Diseases, Jinan Fifth People's Hospital Affiliated to Shandong Second Medical University, Jinan, Shandong, China; 2 Outpatient Department of Jinan Fifth People's Hospital, Affiliated with Shandong Second Medical University, Jinan, Shandong, China; 3 Outpatient Pharmacy, Yingshi Street Community Health Service Center, Jinan, Shandong, China

**Keywords:** ferroptosis, GPx4, lipid peroxidation, neuroprotection, oxidative stress, traditional Chinese medicine, vascular cognitive impairment

## Abstract

**Background:**

Vascular cognitive impairment (VCI) encompasses a spectrum of cognitive deficits arising from cerebrovascular disease, representing a major cause of dementia worldwide. Ferroptosis, a recently identified form of regulated cell death driven by iron-dependent lipid peroxidation, has emerged as a potential contributor to neuronal and glial cell injury in cerebrovascular pathologies, although direct evidence in VCI itself remains limited. Traditional Chinese medicine (TCM) has demonstrated promising neuroprotective effects, with growing evidence suggesting that its therapeutic benefits may partly involve the modulation of ferroptosis pathways.

**Objective:**

This narrative review aims to comprehensively summarize the pathological mechanisms of ferroptosis in VCI, elucidate the molecular crosstalk between ferroptotic cascades and cerebrovascular injury, and highlight the therapeutic potential of TCM interventions targeting ferroptosis in VCI.

**Methods:**

A comprehensive literature search was conducted across PubMed, Web of Science, and CNKI databases for publications from January 2019 to June 2026 (final search performed on 15 April 2026), using predefined terms related to ferroptosis, vascular cognitive impairment, cerebrovascular disease, and traditional Chinese medicine; the full search strategy and evidence-classification scheme are described in Section 1.1.

**Results:**

Preclinical evidence suggests that ferroptosis may contribute to VCI pathogenesis through multiple mechanisms, including iron overload-mediated oxidative damage, glutathione depletion, lipid peroxidation, blood–brain barrier disruption, neuroinflammation, and white matter injury. Several TCM compounds and formulas, including baicalein, salvianolic acid B, gastrodin, astragaloside IV, and Buyang Huanwu Decoction, have demonstrated anti-ferroptotic effects in experimental models through modulation of GPX4, Nrf2/HO-1, System Xc-, and iron metabolism pathways. These preclinical findings provide a preliminary mechanistic basis for the neuroprotective effects of TCM in VCI, although direct clinical evidence is still lacking.

**Conclusion:**

Targeting ferroptosis represents a potential therapeutic strategy for VCI, and TCM offers a rich repository of multi-target, multi-pathway anti-ferroptotic agents. However, most supporting evidence is derived from related cerebrovascular or non-neurological models rather than from VCI itself. Future research should prioritize VCI-specific mechanistic studies, rigorous validation of ferroptosis-specific effects, and clinical translation of TCM-mediated ferroptosis regulation.

## 1 Introduction

Vascular cognitive impairment (VCI) represents a broad category of cognitive disorders attributable to cerebrovascular disease, ranging from mild cognitive deficits to vascular dementia (VaD) (1, 2). As the second most common cause of dementia after Alzheimer’s disease, VCI affects approximately 15–20% of dementia cases globally, with its prevalence projected to rise substantially due to aging populations and increasing cardiovascular risk factors (3). The pathological hallmarks of VCI include cerebral hypoperfusion, white matter lesions, lacunar infarcts, and microbleeds, which collectively compromise the structural and functional integrity of neural circuits essential for cognitive function (4, 5).

Despite the considerable clinical burden of VCI, current therapeutic options remain limited. Existing treatments primarily focus on managing vascular risk factors and cholinesterase inhibitors, which provide only modest symptomatic relief (6). This therapeutic gap underscores the urgent need to identify novel pathological mechanisms and develop targeted interventions for VCI.

Ferroptosis, first coined by Dixon et al. in 2012, is a form of regulated cell death characterized by iron-dependent accumulation of lipid hydroperoxides (7). Unlike apoptosis and necroptosis, ferroptosis is morphologically distinguished by mitochondrial shrinkage, increased membrane density, and reduced mitochondrial cristae (8). The central regulatory axis of ferroptosis involves the cystine/glutamate antiporter System Xc- and the lipid repair enzyme glutathione peroxidase 4 (GPX4), with iron metabolism and lipid peroxidation serving as core executionary mechanisms (9, 10).

The brain is particularly vulnerable to ferroptosis due to its high iron content, abundant polyunsaturated fatty acids (PUFAs), elevated oxygen consumption, and relatively limited antioxidant capacity (11). In the context of cerebrovascular disease, ischemia–reperfusion injury, chronic hypoperfusion, and blood–brain barrier (BBB) disruption create conditions highly conducive to ferroptotic cell death (12, 13). Emerging evidence has implicated ferroptosis in the pathogenesis of multiple neurological conditions, including ischemic stroke, hemorrhagic stroke, traumatic brain injury, and neurodegenerative diseases (14, 15). However, it should be emphasized that direct evidence of ferroptosis in VCI remains limited: most supporting data are extrapolated from related cerebrovascular conditions, such as ischemic and hemorrhagic stroke, or from chronic cerebral hypoperfusion models of VCI (16). Whether ferroptosis acts as a driver of VCI progression or as a secondary consequence of vascular injury has not been definitively established. The specific role of ferroptosis in VCI pathophysiology therefore remains insufficiently elucidated, representing a critical knowledge gap.

Traditional Chinese medicine (TCM), with its philosophical foundation in holistic regulation and multi-target therapeutic approaches, has been used for centuries to treat cerebrovascular diseases and cognitive disorders (17). The TCM theoretical concepts of “blood stasis,” “phlegm obstruction,” and “kidney essence deficiency” offer conceptual parallels to the modern pathological understanding of VCI, encompassing vascular dysfunction, neuroinflammation, and neurodegeneration (18). These parallels should be regarded as conceptual interpretations that generate testable hypotheses, rather than as direct biological equivalences. Recent pharmacological studies have revealed that numerous TCM compounds can modulate ferroptosis-related pathways, including iron homeostasis, lipid peroxidation, and antioxidant defense systems (19, 20).

This review aims to provide a comprehensive synthesis of current knowledge regarding: (1) the molecular mechanisms of ferroptosis; (2) the pathological role of ferroptosis in VCI; and (3) the therapeutic potential of TCM interventions targeting ferroptosis in VCI (Figure 1). By bridging the mechanistic understanding of ferroptosis with the therapeutic arsenal of TCM, this review seeks to identify novel therapeutic strategies and future research directions for VCI management.

**Figure 1:**
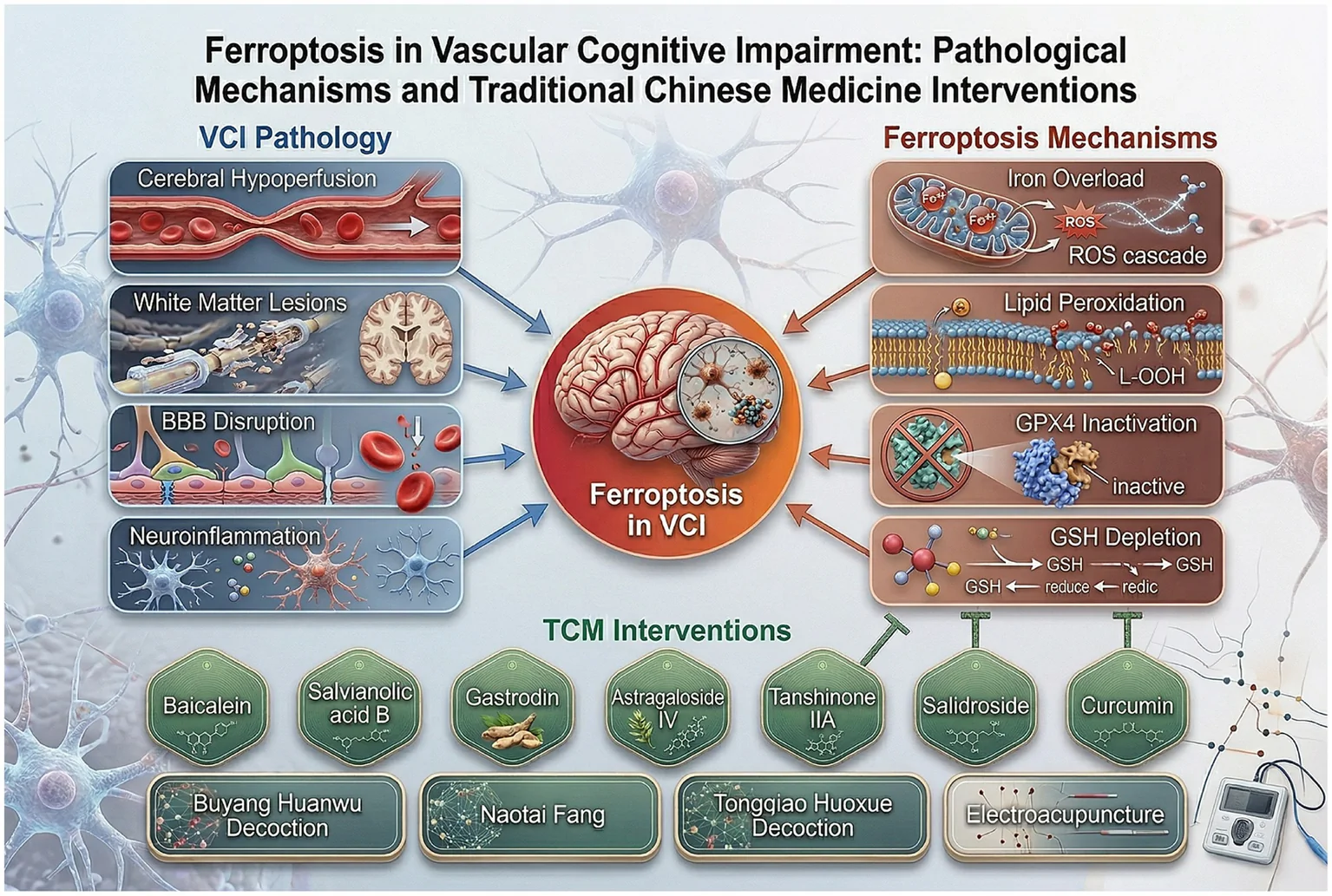
Graphical abstract illustrating the conceptual framework of this review. The central theme “Ferroptosis in VCI” connects three major domains: (left) Pathological features of vascular cognitive impairment including cerebral hypoperfusion, white matter lesions, blood–brain barrier disruption, and neuroinflammation; (right) Core ferroptosis mechanisms including iron overload, lipid peroxidation, and GPX4 inactivation; (bottom) Traditional Chinese medicine interventions targeting ferroptosis, encompassing both single compounds and classical formulas. Arrows indicate the mechanistic connections between domains. Color scheme: Blue for VCI pathology, red for ferroptosis mechanisms, green for TCM interventions.

### 1.1 Literature search and review methodology

This article is intended as a narrative review rather than a systematic review or meta-analysis. A comprehensive literature search was performed in PubMed, Web of Science, and the China National Knowledge Infrastructure (CNKI) for publications from January 2019 to April 2026, with the final search conducted on 15 April 2026. Representative search terms included combinations of “ferroptosis,” “vascular cognitive impairment”, “vascular dementia”, “chronic cerebral hypoperfusion”, “cerebral small vessel disease”, “blood–brain barrier”, “white matter injury”, “traditional Chinese medicine”, and the names of individual compounds and formulas (e.g., “baicalein”, “gastrodin”, “Buyang Huanwu Decoction”). Reference lists of retrieved articles and relevant reviews were additionally screened to identify eligible studies.

Studies were included if they (1) investigated ferroptosis-related mechanisms, biomarkers, or interventions; (2) were conducted in VCI patients, VCI animal models (e.g., chronic cerebral hypoperfusion models), related cerebrovascular or neurological disease models, or relevant *in vitro* systems; and (3) were published in English or Chinese. Editorials, conference abstracts without primary data, and duplicate publications were excluded. Original research articles were prioritized over review articles when supporting specific mechanistic statements.

Titles and abstracts were screened independently by two authors (S.W.Z. and X.J.), and the full texts of potentially eligible articles were assessed independently by the same two authors; any disagreements were resolved through discussion with the third author (S.C.Z.) until consensus was reached.

To evaluate the relevance and strength of the evidence, studies were classified into four tiers: (A) clinical evidence from patients with VCI; (B) direct evidence from established VCI animal models, such as chronic cerebral hypoperfusion models; (C) indirect evidence from related neurological conditions, including ischemic stroke, hemorrhagic stroke, and Alzheimer’s disease; and (D) exploratory evidence from non-neurological disease models or generic cell systems. Throughout this review, and in Table 1, evidence is explicitly labeled according to this hierarchy, and conclusions are drawn accordingly.

**Table 1:** Summary of TCM interventions targeting ferroptosis, classified by experimental model, evidence type, and relevance to VCI.

TCM compound/Formula	Source herb/prescription	Experimental model (disease context)	Ferroptosis-related targets/markers	Key findings	Evidence type	Ferroptosis-specific validation	Relevance to VCI (Tier)	References
Baicalein	Scutellaria baicalensis	tMCAO rats; OGD/R HT22 cells (cerebral ischemia–reperfusion)	12/15-LOX↓, ACSL4↓, GPX4↑, iron chelation	Directly inhibits 12/15-LOX; reduces lipid peroxidation and iron levels; preserves GPX4	Animal + *in vitro*	Identified in a ferroptosis inhibitor library screen (83)	Indirect (C)	(83, 84)
Salvianolic acid B	*Salvia miltiorrhiza*	MCAO rats; OGD/R neurons (cerebral ischemia–reperfusion)	TLR4/MyD88↓, oxidative stress↓	Attenuates inflammation and oxidative stress after CIRI; ferroptosis-specific effects not yet demonstrated	Animal + in vitro	Not performed	Indirect (C)	(85, 86)
Gastrodin	Gastrodia elata	BCCAO VaD rats; hypoxia-injured HT22 cells; ischemic stroke models	Nrf2/Keap1-GPX4↑, xCT↑, ACSL4/LPCAT3↓	Decreases Fe^2+^ and MDA; increases GSH; improves learning and memory in VaD rats	Animal + in vitro	Comparison with ferrostatin-1; erastin/ML385 pathway modulation (68)	Direct (B)	(68, 87)
Astragaloside IV	Astragalus membranaceus	MCAO rats (cerebral ischemia–reperfusion); neuronal cells	P62/Keap1/Nrf2↑, SLC7A11/GPX4↑	Promotes P62-mediated Keap1 degradation and Nrf2 activation; protects neuronal mitochondria	Animal + in vitro	Pharmacological pathway modulation (88)	Indirect (C)	(88, 89)
Tanshinone IIA	*Salvia miltiorrhiza*	MCAO/R rats; OGD/R SH-SY5Y cells (cerebral ischemia–reperfusion)	miR-449a↑/ACSL4↓	Suppresses ACSL4 via miR-449a; reduces neuronal ferroptosis and infarct volume	Animal + in vitro	miR-449a modulation (90)	Indirect (C)	(90)
Salidroside	*Rhodiola rosea*	pMCAO rats; renal ischemia–reperfusion model (non-neurological)	PI3K/AKT; inflammation↓	Reduces brain injury after pMCAO; anti-ferroptotic effect demonstrated only in renal ischemia–reperfusion	Animal	Not performed in neurological models	Exploratory (D)	(91, 92)
Curcumin	*Curcuma longa*	SAH rats; rhabdomyolysis-associated renal injury (non-neurological)	Nrf2/HO-1/GPX4↑, iron chelation	Attenuates neuroinflammation and neuronal ferroptosis after SAH	Animal	Marker-based	Indirect (C)	(93, 94)
Resveratrol	*Polygonum cuspidatum*	Myocardial ischemia–reperfusion (non-neurological)	SIRT1/Nrf2↑, GPX4↑, iron accumulation↓	Attenuates ferroptosis after myocardial ischemia–reperfusion	Animal	Marker-based	Exploratory (D)	(95)
Puerarin	*Pueraria lobata*	Glutamate-injured HT22 cells (in vitro)	Nrf2/GPX4↑	Protects neuronal cells against glutamate-induced ferroptosis in vitro	In vitro	Marker-based	Indirect (C)	(82)
Icariin	Epimedium	Glucocorticoid-induced bone deterioration (non-neurological)	GPX4↑, FTH1↑, NCOA4↓	Inhibits NCOA4-mediated ferritinophagy in a bone deterioration model	Animal	Marker-based	Exploratory (D)	(96)
Ginsenoside Rg1	*Panax ginseng*	Sepsis-induced acute kidney injury (non-neurological)	PI3K/Akt/Nrf2↑, GPX4↑, SLC7A11↑	Inhibits ferroptosis in sepsis-induced acute kidney injury	Animal	Marker-based	Exploratory (D)	(97)
Buyang Huanwu Decoction	Classical formula	MCAO mice; OGD/R HT22 cells (ischemic stroke)	Nrf2/GPX4/SLC7A11↑, ACSL4↓	Activates the Nrf2/GPX4 pathway; reduces iron overload and infarct volume	Animal + in vitro	Marker-based	Indirect (C)	(98–100)
Naotai Fang	Clinical prescription	CIRI rats; VCI models (as summarized in reviews)	HSP90/GCN2/ATF4↓, PI3K/Akt/Nrf2↑	Reported to inhibit ferroptosis and necroptosis and to reduce BBB permeability; mechanisms require confirmation in original studies	Animal (reported)	Unclear (secondary sources)	Reported, indirect (B-C)	(17, 18)
Tongqiao Huoxue Decoction	Classical formula	tMCAO rats; OGD/R PC12 cells (as summarized in reviews)	GSH-GPX4↑, FTH1↑; reported ACSL4 ubiquitination↑	Reported to reduce iron deposition and lipid peroxidation; the ACSL4 ubiquitination mechanism requires verification in original studies	Animal (reported)	Unclear (secondary sources)	Reported, indirect (B-C)	(17, 18)
Electroacupuncture	Acupoints: GV20, GV26, SP6, PC6 (101); GV20, GV14, LI11, PC6 (102)	MCAO/R rats (cerebral ischemia)	Nrf2/SLC7A11/GPX4↑, Fe^2+^↓, ROS/MDA↓	Promotes Nrf2 nuclear translocation; reduces iron accumulation and oxidative stress	Animal	Reversal by Nrf2 inhibition (102)	Indirect (C)	(101, 102)

BCCAO, bilateral common carotid artery occlusion; CIRI, cerebral ischemia–reperfusion injury; MCAO, middle cerebral artery occlusion; OGD/R, oxygen–glucose deprivation/reoxygenation; pMCAO, permanent middle cerebral artery occlusion; SAH, subarachnoid hemorrhage; tMCAO, transient middle cerebral artery occlusion; VaD, vascular dementia. Evidence tiers: A, clinical evidence from VCI patients; B, direct evidence from VCI animal models; C, indirect evidence from related neurological models; D, exploratory evidence from non-neurological models (see Section 1.1).

## 2 Overview of ferroptosis mechanisms

This section summarizes the core molecular machinery of ferroptosis. It should be noted that these mechanisms have been delineated largely in cancer and non-neural systems; where evidence from cerebrovascular or VCI models is available, this is explicitly indicated, and the distinction between general ferroptosis biology and pathways directly investigated in VCI models is maintained throughout.

### 2.1 Iron metabolism and ferroptosis

Iron homeostasis plays a central role in ferroptosis execution. Under physiological conditions, iron metabolism is tightly regulated through a coordinated network involving transferrin receptor 1 (TfR1)-mediated iron uptake, ferritin-dependent iron storage, and ferroportin (FPN)-mediated iron export (21). In the brain, iron is essential for numerous physiological processes, including neurotransmitter synthesis, myelination, and mitochondrial respiration (22).

During ferroptosis, dysregulated iron metabolism leads to the accumulation of labile iron pool (LIP), which catalyzes the Fenton reaction (Fe^2+^ + H₂O₂ → Fe^3+^ + OH^−^ + •OH), generating highly reactive hydroxyl radicals that initiate lipid peroxidation (23). Nuclear receptor coactivator 4 (NCOA4)-mediated ferritinophagy, a selective autophagic degradation of ferritin, further amplifies intracellular free iron levels and promotes ferroptosis (24). Additionally, iron regulatory proteins (IRP1 and IRP2) modulate the expression of iron metabolism-related genes at the post-transcriptional level, fine-tuning cellular iron availability (25).

In the context of cerebrovascular disease, iron overload occurs through multiple mechanisms: (1) hemoglobin degradation following microbleeds releases heme-bound iron; (2) BBB disruption permits extravasation of transferrin-bound iron; and (3) ischemia-induced acidosis promotes iron release from ferritin (26, 27). These pathological iron sources significantly elevate the vulnerability of neural cells to ferroptosis.

### 2.2 Lipid peroxidation: the executioner of ferroptosis

Lipid peroxidation of polyunsaturated fatty acid-containing phospholipids (PUFA-PLs) constitutes the hallmark event of ferroptosis (28). The process involves three critical steps: (1) incorporation of PUFAs into membrane phospholipids by acyl-CoA synthetase long-chain family member 4 (ACSL4) and lysophosphatidylcholine acyltransferase 3 (LPCAT3); (2) oxidation of PUFA-PLs by lipoxygenases (LOXs) or through iron-catalyzed non-enzymatic mechanisms; and (3) propagation of lipid radical chain reactions (29, 30).

ACSL4 has been identified as a critical determinant of ferroptosis sensitivity by catalyzing the esterification of arachidonic acid (AA) and adrenalic acid (AdA) into phosphatidylethanolamine (PE), generating the primary substrates for lipid peroxidation (31). Conversely, the suppression of ACSL4 expression confers resistance to ferroptosis. The 15-lipoxygenase (15-LOX)/phosphatidylethanolamine-binding protein 1 (PEBP1) complex has been identified as a key enzymatic driver of ferroptotic lipid peroxidation, selectively oxidizing arachidonoyl-PE species (32).

Beyond the canonical ACSL4/LPCAT3-PUFA axis, several emerging layers of lipid regulation modulate ferroptosis sensitivity. Monounsaturated fatty acids (MUFAs), such as oleic acid, are incorporated into membrane phospholipids via ACSL3 and competitively displace oxidizable PUFAs, thereby promoting a ferroptosis-resistant cell state (33). Lipid droplets act as buffering organelles that sequester PUFAs and limit their availability for peroxidation, whereas lipophagy-mediated degradation of lipid droplets releases lipid substrates that can fuel ferroptosis (10, 34). In addition, the abundance and subcellular distribution of coenzyme Q and of endogenous radical-trapping antioxidants (including CoQ10, tetrahydrobiopterin, and vitamin E) determine the threshold at which lipid radical chain reactions are terminated (10, 35, 36). These emerging concepts remain largely unexplored in cerebrovascular disease and warrant investigation in VCI models.

The accumulation of lipid hydroperoxides (LOOH) in cellular membranes ultimately compromises membrane integrity, leading to cell death. The specific lipid peroxidation products, including 4-hydroxynonenal (4-HNE) and malondialdehyde (MDA), serve as both effectors and biomarkers of ferroptosis (37).

### 2.3 GPX4 and the glutathione antioxidant system

Glutathione peroxidase 4 (GPX4) serves as the master regulator of ferroptosis by catalyzing the reduction of lipid hydroperoxides to non-toxic lipid alcohols, thereby preventing the accumulation of lethal lipid peroxidation products (38). GPX4 utilizes reduced glutathione (GSH) as its essential cofactor, establishing the GSH-GPX4 axis as the primary defense against ferroptosis (39).

GSH biosynthesis depends on the availability of cysteine, which is predominantly imported into cells via System Xc- (encoded by SLC7A11 and SLC3A2) as cystine in exchange for intracellular glutamate (40). Pharmacological inhibition of System Xc- by compounds such as erastin depletes intracellular cysteine and GSH, subsequently inactivating GPX4 and triggering ferroptosis (7). Alternatively, direct inhibition of GPX4 by RSL3 and related compounds bypasses the GSH requirement and directly induces ferroptosis (41).

The transcription factor nuclear factor erythroid 2-related factor 2 (Nrf2) serves as a master regulator of antioxidant gene expression, controlling the transcription of numerous ferroptosis-related genes, including SLC7A11, GPX4, heme oxygenase-1 (HO-1), ferritin heavy chain (FTH1), and NAD(P)H quinone dehydrogenase 1 (NQO1) (42, 43). Under oxidative stress, Nrf2 dissociates from its cytoplasmic inhibitor Keap1, translocates to the nucleus, and activates the antioxidant response element (ARE)-driven gene transcription, providing comprehensive protection against ferroptosis (44).

### 2.4 GPX4-independent ferroptosis defense pathways

Recent research has uncovered several GPX4-independent anti-ferroptotic mechanisms that provide additional layers of protection:

*FSP1-CoQ10 pathway*: Ferroptosis suppressor protein 1 (FSP1, also known as DHODH-independent CoQ oxidoreductase) functions as an oxidoreductase that reduces coenzyme Q10 (CoQ10, ubiquinone) to ubiquinol (CoQ10H2), which acts as a lipid-soluble radical-trapping antioxidant (RTA) to suppress lipid peroxidation independent of GPX4 (35, 45).

*DHODH pathway*: Dihydroorotate dehydrogenase (DHODH), localized in the inner mitochondrial membrane, reduces CoQ to CoQH2 in the mitochondria, providing a parallel defense mechanism against mitochondrial lipid peroxidation (46).

*GCH1-BH4 pathway*: GTP cyclohydrolase 1 (GCH1) catalyzes the biosynthesis of tetrahydrobiopterin (BH4), a potent radical-trapping antioxidant that selectively protects phospholipids containing two PUFA tails from oxidative degradation (36).

*MBOAT1/2 pathway*: Membrane-bound O-acyltransferase domain-containing 1 and 2 (MBOAT1/2) have been recently identified as sex hormone-regulated ferroptosis suppressors that remodel the cellular phospholipid composition to reduce ferroptosis sensitivity (47).

*Mitochondrial regulation of ferroptosis*: Mitochondria are increasingly recognized as central modulators of ferroptosis beyond the DHODH pathway. Electron transport chain (ETC) activity, particularly at complexes I and III, generates mitochondrial reactive oxygen species that promote lipid peroxidation, and mitochondrial glutaminolysis fuels this process under cysteine-deprived conditions (48). Mitochondrial lipid remodeling and the mitochondrial iron pool, governed by mitoferrin-2-mediated iron import, mitochondrial ferritin storage, and iron–sulfur cluster biogenesis, further shape ferroptosis sensitivity (48). These mechanisms are highly relevant to cerebrovascular injury, in which chronic hypoperfusion and ischemia–reperfusion disrupt mitochondrial integrity and ETC function, potentially lowering the ferroptosis threshold of neural cells (48, 49).

These complementary defense systems highlight the complexity of ferroptosis regulation and provide additional therapeutic targets for ferroptosis modulation (Figure 2).

**Figure 2:**
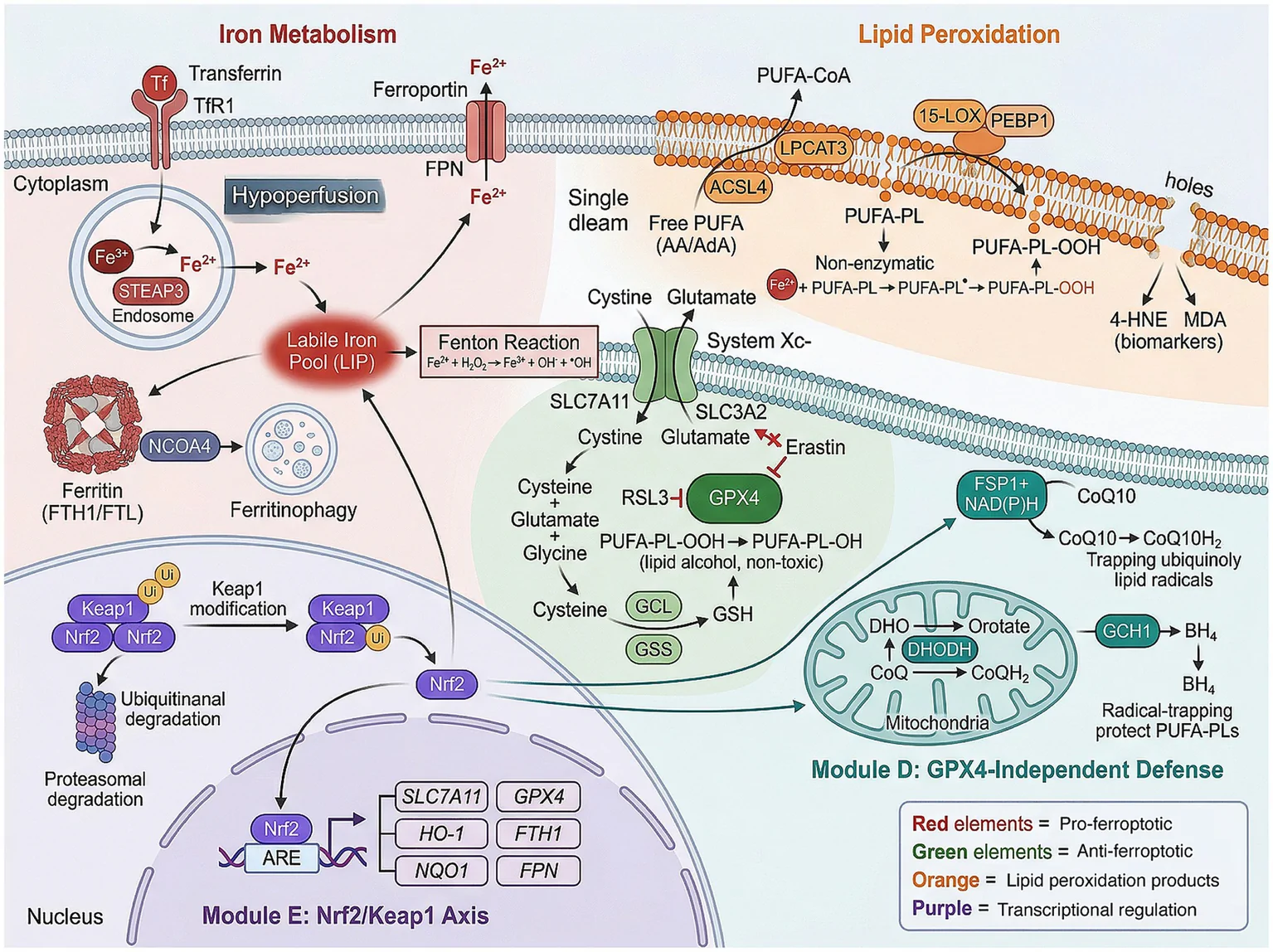
Molecular mechanisms of ferroptosis. The diagram depicts the core regulatory pathways of ferroptosis: **(A)** Iron metabolism—TfR1-mediated iron uptake, endosomal Fe^2+^ release into the labile iron pool (LIP), Fenton reaction generating hydroxyl radicals, NCOA4-mediated ferritinophagy, and ferroportin-mediated iron export; **(B)** Lipid peroxidation—ACSL4/LPCAT3-dependent incorporation of PUFAs into membrane phospholipids, LOX-catalyzed and non-enzymatic oxidation generating PUFA-PL-OOH, and membrane rupture; **(C)** GPX4 antioxidant defense—System Xc- (SLC7A11)-mediated cystine import, GSH biosynthesis, and GPX4-catalyzed reduction of LOOH to LOH; **(D)** GPX4-independent defense systems—FSP1/CoQ10 pathway, DHODH/CoQH2 pathway, and GCH1/BH4 pathway; **(E)** Nrf2/Keap1 regulatory axis controlling transcription of SLC7A11, GPX4, HO-1, FTH1, and NQO1. Pro-ferroptotic factors are shown in red; anti-ferroptotic factors are shown in green.

### 2.5 Ferroptosis crosstalk with other regulated cell death pathways

Ferroptosis does not operate in isolation but engages in extensive crosstalk with other forms of regulated cell death. Autophagy, particularly ferritinophagy (NCOA4-mediated) and lipophagy, can promote ferroptosis by increasing intracellular free iron and lipid substrates (34). Conversely, chaperone-mediated autophagy of GPX4 has been identified as a mechanism linking autophagy to ferroptosis execution (50).

The p53 tumor suppressor regulates ferroptosis through both transcription-dependent and -independent mechanisms, including suppression of SLC7A11 expression and promotion of ALOX12-mediated lipid peroxidation (51). Additionally, the AMPK signaling pathway modulates ferroptosis sensitivity through its effects on lipid metabolism and energy homeostasis (52). The key regulatory pathways of ferroptosis and their specific relevance to VCI pathogenesis are summarized in Table 2.

**Table 2:** Key regulatory pathways of ferroptosis and their roles in VCI.

Pathway/mechanism	Key molecules	Role in ferroptosis	Relevance to VCI	References
Iron metabolism	TfR1, Ferritin, FPN, NCOA4, IRP1/2	Iron uptake, storage, export, and ferritinophagy regulate labile iron pool (LIP); excess LIP catalyzes Fenton reaction to generate lipid radicals	CCH upregulates TfR1 and downregulates FPN; microbleeds release heme iron; iron accumulation in basal ganglia and white matter correlates with cognitive decline	(21–27, 59)
Lipid peroxidation	ACSL4, LPCAT3, LOXs, PEBP1	ACSL4/LPCAT3 incorporate PUFAs into membrane PLs; LOXs and Fenton chemistry oxidize PUFA-PLs to lethal lipid hydroperoxides (LOOH)	Oligodendrocytes enriched in PUFA-PLs are highly susceptible; lipid peroxidation disrupts synaptic membranes and myelin sheaths	(28–32, 37)
GSH-GPX4 axis	System Xc- (SLC7A11), GSH, GPX4	System Xc- imports cystine for GSH synthesis; GPX4 reduces LOOH to non-toxic LOH; inhibition of either triggers ferroptosis	GSH depletion and GPX4 downregulation have been documented in a BCCAO model of vascular dementia; broader VCI-specific data remain limited	(38–41, 68)
Nrf2/Keap1 signaling	Nrf2, Keap1, HO-1, NQO1, FTH1	Nrf2 transcriptionally activates SLC7A11, GPX4, HO-1, FTH1, and NQO1; master regulator of antioxidant defense against ferroptosis	Nrf2 activation is a primary therapeutic target for most TCM anti-ferroptotic compounds in VCI	(42–44)
FSP1-CoQ10 pathway	FSP1 (AIFM2), CoQ10, NAD(P)H	FSP1 reduces CoQ10 to CoQ10H2, a lipid-soluble radical-trapping antioxidant; GPX4-independent defense	Provides parallel defense in neurons and endothelial cells; relevance to VCI remains to be tested	(35, 45)
DHODH pathway	DHODH, CoQ, CoQH2	DHODH reduces CoQ to CoQH2 in mitochondrial inner membrane; mitigates mitochondrial lipid peroxidation	Mitochondrial dysfunction in hypoperfused brain amplifies need for DHODH defense	(46)
Mitochondrial metabolism	ETC complexes I/III, DHODH, mitoferrin-2, mitochondrial ferritin	Mitochondrial ROS, electron transport chain activity, and mitochondrial iron handling modulate lipid peroxidation and the ferroptosis threshold	Chronic hypoperfusion disrupts mitochondrial integrity, potentially lowering the ferroptosis threshold of neural cells	(46, 48)
GCH1-BH4 pathway	GCH1, BH4	GCH1 synthesizes BH4, a radical-trapping antioxidant that selectively protects PUFA-PLs from peroxidation	Complements GPX4 and FSP1 in protecting neural membrane lipids	(36)
Autophagy-ferroptosis crosstalk	NCOA4, LC3, BECN1, CMA	Ferritinophagy (NCOA4) releases iron; lipophagy provides lipid substrates; CMA degrades GPX4; all promote ferroptosis	Autophagy dysregulation in VCI may amplify ferroptosis via ferritinophagy and GPX4 degradation	(34, 50)

### 2.6 Criteria for demonstrating ferroptosis: association versus causation

A critical issue in interpreting the literature is that commonly measured ferroptosis markers, including decreased GPX4 or GSH and elevated ROS, MDA, 4-HNE, or iron levels, are not by themselves sufficient to establish ferroptotic cell death, as they may equally reflect general oxidative stress or non-specific tissue injury (9, 10).

Rigorous demonstration of ferroptosis requires converging evidence: (1) iron dependency, shown by rescue with iron chelators; (2) lipid peroxidation dependency, shown by rescue with ferroptosis-specific inhibitors (e.g., ferrostatin-1, liproxstatin-1) but not by inhibitors of apoptosis, necroptosis, or pyroptosis; (3) genetic evidence from manipulation of ferroptosis regulators (e.g., GPX4, ACSL4, FSP1, SLC7A11); and (4) compatible morphological features, such as mitochondrial shrinkage on electron microscopy (10, 53).

Throughout this review, we therefore distinguish between ferroptosis-associated changes, which constitute the majority of the available VCI-related evidence, and experimentally validated ferroptosis-mediated causal effects. Statements supported only by marker changes should be interpreted as associations rather than proof of ferroptosis.

## 3 Ferroptosis in the pathogenesis of VCI

The evidence linking ferroptosis to VCI varies considerably in directness. In line with the hierarchy defined in Section 1.1, the following subsections distinguish among (A) clinical evidence from VCI patients, (B) direct evidence from VCI animal models such as chronic cerebral hypoperfusion, (C) indirect evidence from related neurological disease models, and (D) exploratory evidence from non-neurological models.

### 3.1 VCI: classification, epidemiology, and pathological features

Vascular cognitive impairment encompasses a heterogeneous group of cognitive disorders caused by cerebrovascular disease, ranging from vascular mild cognitive impairment (VaMCI) to vascular dementia (VaD) (1). The major subtypes include post-stroke dementia, subcortical ischemic vascular dementia (SIVD), multi-infarct dementia, and mixed dementia with concurrent neurodegenerative pathology (54). The neuropathological hallmarks of VCI include white matter hyperintensities (WMH), lacunar infarcts, cerebral microbleeds, and diffuse cortical–subcortical atrophy (55).

The pathophysiology of VCI involves a complex interplay of cerebral hypoperfusion, oxidative stress, neuroinflammation, BBB disruption, and synaptic dysfunction (56). Chronic cerebral hypoperfusion, resulting from arteriosclerosis, arteriolosclerosis, or cerebral amyloid angiopathy, leads to sustained ischemic injury of the white matter and deep gray matter structures, disrupting the neural networks critical for cognitive function (49).

### 3.2 Iron dysregulation in cerebrovascular disease and VCI

Iron accumulation in the brain is a consistent finding in cerebrovascular diseases associated with VCI. Cerebral microbleeds, a hallmark feature of small vessel disease, release hemoglobin-derived iron into the perivascular space, creating focal regions of iron overload (57). The degradation of hemoglobin by heme oxygenase-1 (HO-1) releases free iron, carbon monoxide, and biliverdin, with the liberated iron capable of catalyzing Fenton reactions and driving ferroptosis (58).

Magnetic resonance imaging (MRI) studies have demonstrated increased iron deposition in the basal ganglia, thalamus, and white matter of patients with VCI, correlating with cognitive decline severity (59). Quantitative susceptibility mapping (QSM) has revealed that iron accumulation in strategic brain regions is associated with, and may precede, overt cognitive impairment (60). However, such imaging associations do not establish ferroptosis as the causal mechanism: cerebral iron deposition also increases with aging and inflammation, and alternative iron-mediated mechanisms of injury cannot be excluded (22, 60).

Furthermore, chronic cerebral hypoperfusion impairs the iron regulatory mechanisms in the brain. Ischemic conditions upregulate TfR1 expression while downregulating ferroportin, creating an imbalance favoring intracellular iron accumulation (27). The resulting iron overload amplifies oxidative stress, lipid peroxidation, and ferroptotic cell death in neurons, oligodendrocytes, and endothelial cells.

Importantly, iron deposition alone does not equate to ferroptotic activation. It is the redox-active labile iron pool (LIP), rather than total tissue iron, that fuels Fenton chemistry and lipid peroxidation (23). The size of the LIP is dynamically controlled by the balance between TfR1-mediated uptake, ferritin sequestration, NCOA4-mediated ferritinophagy (which releases stored iron), and ferroportin-mediated export (21, 24). Mitochondrial iron metabolism, including mitoferrin-2-mediated mitochondrial import and mitochondrial ferritin storage, adds a further regulatory layer, linking iron handling to electron transport chain activity and mitochondrial ROS production (48). During VCI development, chronic hypoperfusion may simultaneously increase iron influx (TfR1 upregulation), reduce iron efflux (ferroportin downregulation), and enhance ferritinophagy, jointly expanding the LIP and enabling iron-dependent lipid oxidation (27, 49).

### 3.3 Ferroptosis and blood–brain barrier disruption

The BBB, composed of brain endothelial cells, pericytes, and astrocytic endfeet, plays a critical role in maintaining cerebral homeostasis (61). Ferroptosis of brain endothelial cells has been identified as a novel mechanism of BBB disruption in cerebrovascular disease (62).

Endothelial cell ferroptosis is triggered by iron overload and oxidative stress during ischemia–reperfusion injury, leading to the degradation of tight junction proteins (claudin-5, occludin, and ZO-1) and increased paracellular permeability (63). This BBB breakdown creates a vicious cycle: increased permeability allows extravasation of blood-borne iron and inflammatory mediators into the brain parenchyma, further exacerbating iron overload and ferroptotic cell death (14).

In experimental models, ferroptosis of brain microvascular endothelial cells has been shown to mediate BBB disruption after traumatic brain injury, and treatment with ferrostatin-1 reduced endothelial cell death and preserved BBB integrity (64). Although this evidence derives from a traumatic rather than a vascular model, it suggests that endothelial ferroptosis may contribute to BBB dysfunction in cerebrovascular disease and potentially to VCI progression; direct validation in VCI models is still required.

### 3.4 Ferroptosis in white matter injury and demyelination

White matter injury is the predominant neuropathological feature of subcortical VCI, characterized by demyelination, oligodendrocyte loss, and axonal degeneration (65). Oligodendrocytes, the myelinating cells of the central nervous system, are particularly susceptible to ferroptosis due to their high iron content required for myelination and their abundant expression of PUFA-containing lipids (66).

Ferroptosis of oligodendrocytes and oligodendrocyte precursor cells (OPCs) has been demonstrated in the cuprizone demyelination model, in which it impairs myelin maintenance (67); whether chronic cerebral hypoperfusion triggers oligodendrocyte ferroptosis in VCI models such as bilateral common carotid artery stenosis (BCAS) remains to be directly tested. Notably, in the bilateral common carotid artery occlusion (BCCAO) rat model of vascular dementia, hippocampal ferroptosis markers (elevated Fe^2+^ and MDA; decreased GSH and GPX4) are significantly altered, and the ferroptosis inhibitor ferrostatin-1 ameliorates cognitive deficits, providing direct preclinical evidence of ferroptosis involvement in a VCI model (68). In contrast, astrocyte ferroptosis driven by NOX4-mediated oxidative stress has so far been documented mainly in an Alzheimer’s disease context (69), constituting indirect evidence for VCI.

The susceptibility of oligodendrocytes to ferroptosis is further amplified by their limited antioxidant reserve and dependence on transferrin-delivered iron for myelin synthesis (70). This inherent vulnerability positions oligodendrocyte ferroptosis as a central mechanism linking chronic hypoperfusion to the white matter pathology characteristic of VCI.

### 3.5 Neuroinflammation and ferroptosis in VCI

Ferroptosis and neuroinflammation are intimately interconnected in VCI pathogenesis. Ferroptotic cells release damage-associated molecular patterns (DAMPs), including high-mobility group box 1 (HMGB1), oxidized phospholipids, and prostaglandins, which activate innate immune responses through pattern recognition receptors (PRRs) on microglia and astrocytes (71, 72).

Microglial activation following ferroptotic stimuli leads to the production of pro-inflammatory cytokines (TNF-α, IL-1β, and IL-6), reactive oxygen species (ROS), and nitric oxide (NO), which create a neuroinflammatory milieu that further promotes ferroptosis in neighboring neurons and glial cells (73). This feedforward loop between ferroptosis and neuroinflammation has been demonstrated largely in stroke and neurodegenerative models; in VCI it remains a proposed mechanism that may amplify tissue damage and contribute to disease progression.

The NLRP3 inflammasome pathway has been proposed as a potential link between ferroptosis and neuroinflammation. Iron overload and lipid peroxidation products activate the NLRP3 inflammasome, leading to caspase-1-dependent maturation and release of IL-1β and IL-18 (74). Conversely, NLRP3-derived inflammatory mediators have been suggested to promote ferroptosis by suppressing GPX4 expression and enhancing iron uptake, although this feedback has not yet been validated in VCI models.

Additionally, ferroptosis-induced release of oxidized phosphatidylethanolamines (oxPE) serves as an “eat-me” signal for phagocytic clearance, bridging ferroptotic cell death with the inflammatory resolution process (75).

### 3.6 Ferroptosis and synaptic dysfunction in VCI

Synaptic dysfunction is a critical substrate of cognitive impairment in VCI. Emerging evidence, derived predominantly from neurodegenerative disease models, suggests that ferroptosis may contribute to synaptic pathology through multiple mechanisms (76). Lipid peroxidation of synaptic membranes alters the biophysical properties of the lipid bilayer, impairing neurotransmitter receptor trafficking and synaptic vesicle dynamics (77).

Iron accumulation in presynaptic terminals disrupts calcium homeostasis and neurotransmitter release, while oxidative damage to postsynaptic density proteins compromises synaptic plasticity (78). Alterations in synaptic proteins (decreased PSD-95 and synaptophysin) and oxidative damage to synaptic components have been reported in cerebrovascular and neurodegenerative models, but VCI-specific studies directly linking ferroptosis to synaptic failure are currently lacking (79).

Furthermore, glutamate excitotoxicity, a well-established mechanism in VCI, is mechanistically linked to ferroptosis through System Xc- activity. The exchange of intracellular glutamate for extracellular cystine by System Xc- contributes to extracellular glutamate accumulation, potentially exacerbating excitotoxic neuronal injury while simultaneously maintaining cellular GSH levels (80). The comprehensive pathological cascade of ferroptosis in VCI is illustrated in Figure 3.

**Figure 3:**
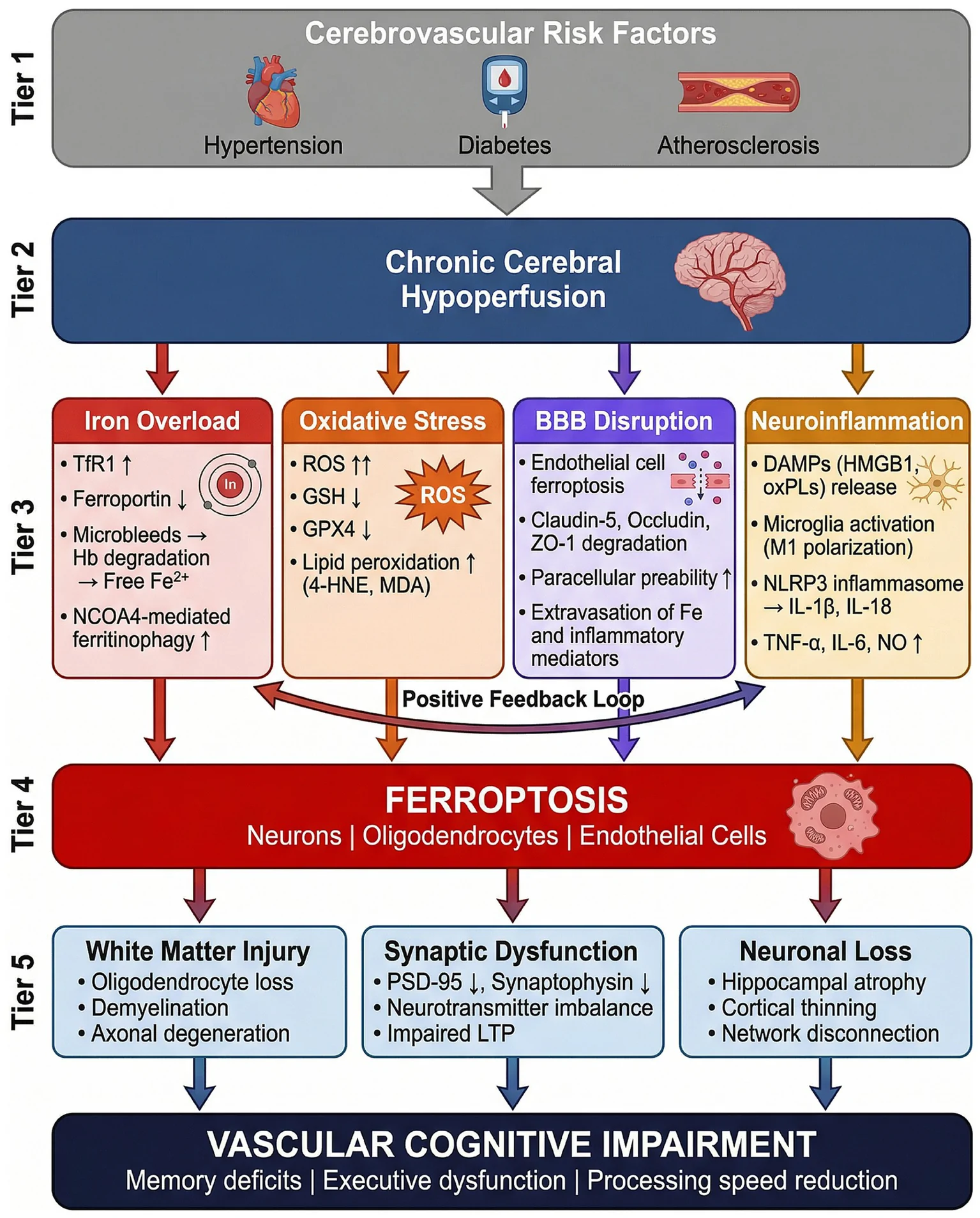
Pathological mechanisms of ferroptosis in vascular cognitive impairment. The flowchart illustrates the pathological cascade from cerebrovascular risk factors (hypertension, diabetes, atherosclerosis) through chronic cerebral hypoperfusion to four parallel ferroptosis-promoting pathways: (1) iron overload (TfR1 upregulation, FPN downregulation, microbleed-derived hemoglobin degradation); (2) oxidative stress (ROS accumulation, GSH depletion, GPX4 inactivation); (3) blood–brain barrier disruption (endothelial cell ferroptosis, tight junction protein degradation); (4) neuroinflammation (DAMP release, microglial activation, NLRP3 inflammasome engagement). These converge upon ferroptotic death of neurons, oligodendrocytes, and endothelial cells, leading to white matter injury, synaptic dysfunction, and neuronal loss, ultimately resulting in vascular cognitive impairment. Bidirectional arrows highlight the proposed positive feedback loop between ferroptosis and neuroinflammation. The loop is marked as proposed in the figure, and the evidence tier of each pathway is classified according to Section 1.1.

### 3.7 Cell-type-specific ferroptosis regulation in VCI

Neural cell populations differ markedly in iron handling, lipid composition, and antioxidant capacity, and therefore exhibit distinct ferroptosis vulnerabilities (9). Oligodendrocytes combine an exceptionally high iron content, required for myelin synthesis, with PUFA-rich membranes and limited antioxidant reserves, rendering them particularly susceptible during chronic hypoperfusion (67, 70). Neurons, enriched in PUFA-containing phospholipids and highly dependent on GPX4-mediated defense, undergo ferroptosis when GPX4 activity or cysteine supply is compromised (7, 38).

Microglia display cell-state-dependent ferroptosis sensitivity: SEC24B regulates microglial ferroptosis, and ferroptotic microglia accumulate in aging human white matter, contributing to neurodegeneration and white matter injury (66, 73). Astrocytes are comparatively resistant owing to robust Nrf2-driven antioxidant defenses, but NOX4-derived oxidative stress can overcome this protection and trigger astrocyte ferroptosis, as shown in an Alzheimer’s disease model (69). Cerebrovascular endothelial cells are vulnerable to iron-overload- and ischemia-associated ferroptosis, with direct consequences for tight junction integrity and BBB permeability (64).

These cell-type-specific features have two implications for VCI. First, therapeutic windows and targets may differ among cell types, arguing for cell-directed delivery strategies. Second, intercellular coupling, for example astrocyte- and microglia-derived inflammatory or lipid mediators acting on oligodendrocytes and neurons, means that ferroptosis in one population can propagate injury to others (72, 73). Systematic mapping of ferroptosis susceptibility across neural cell types in VCI models, using single-cell and spatial approaches, is an important future direction (see Section 5.2).

## 4 Traditional Chinese medicine interventions targeting ferroptosis in VCI

### 4.1 Theoretical framework: TCM perspectives on VCI and ferroptosis

In TCM theory, VCI is primarily attributed to the pathological mechanisms of “blood stasis obstructing collaterals”, “phlegm-dampness clouding the orifices”, and “kidney essence deficiency failing to nourish the brain” (80). These traditional concepts offer conceptual parallels to modern pathological understanding: blood stasis may be conceptually mapped onto vascular dysfunction and thrombosis; phlegm-dampness onto neuroinflammation and metabolic derangement; and kidney essence deficiency onto neurodegenerative processes (81). We emphasize that these mappings are interpretive frameworks intended to guide hypothesis generation, not established biological equivalences.

The ferroptosis-related pathological features—iron overload, lipid peroxidation, and oxidative stress—can be conceptualized within the TCM framework as manifestations of “toxic stasis” and “turbid phlegm generating heat” (81). This theoretical convergence provides a rationale for exploring TCM interventions that target ferroptosis in VCI.

The multi-component, multi-target nature of TCM formulations is particularly well-suited for addressing the complex pathological network of ferroptosis, which involves the simultaneous dysregulation of iron metabolism, lipid peroxidation, and antioxidant defense systems (82). The anti-ferroptotic targets and mechanisms of TCM interventions in VCI are summarized in Figure 4.

**Figure 4:**
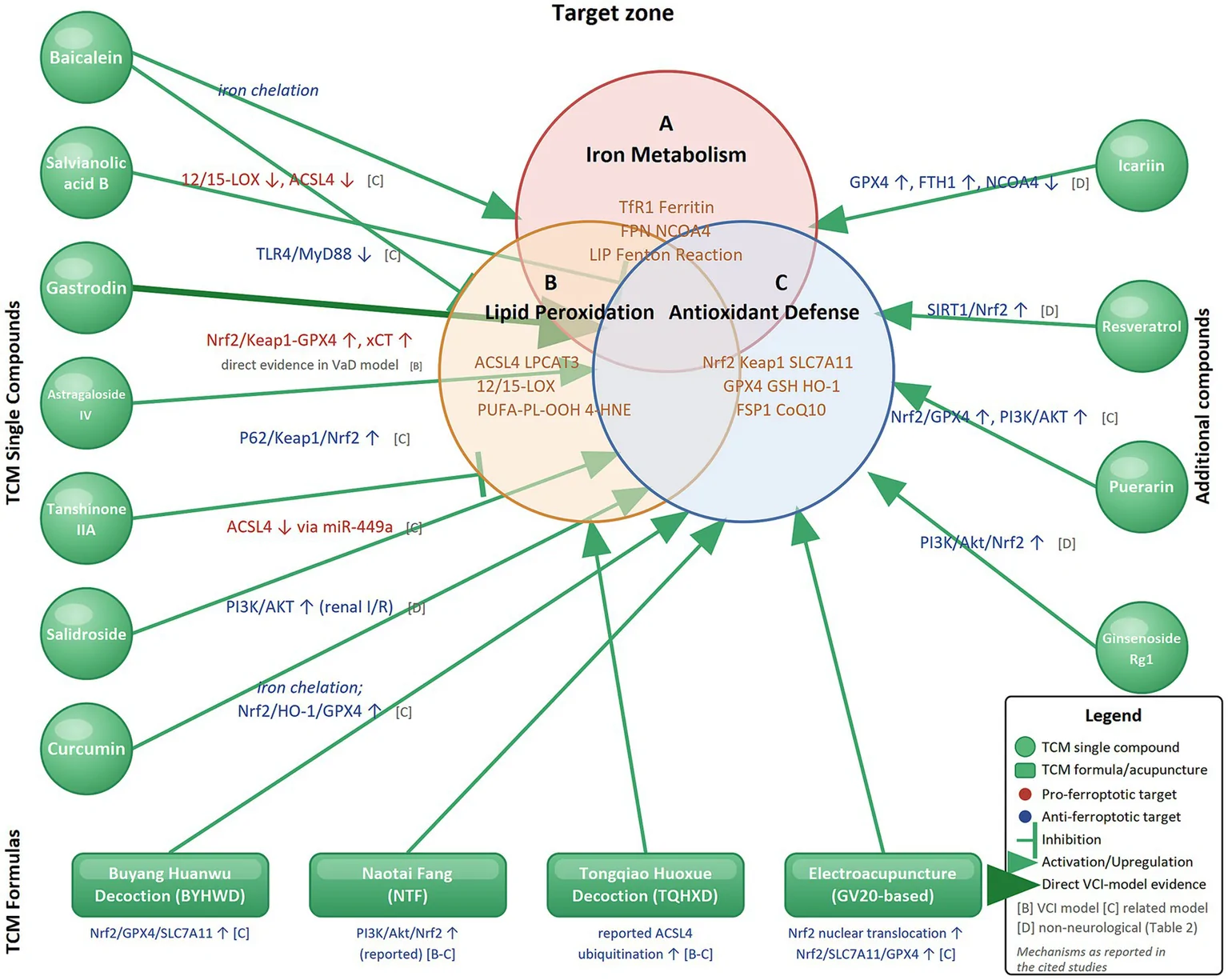
Summary of traditional Chinese medicine interventions targeting ferroptosis in VCI. The schematic organizes TCM anti-ferroptotic agents around three core mechanistic targets: **(A)** Iron metabolism — Curcumin (iron chelation), Astragaloside IV (NCOA4-mediated ferritinophagy inhibition), Salvianolic acid B (ferroportin upregulation); **(B)** Lipid peroxidation — Baicalein (12/15-LOX inhibition), Curcumin (ACSL4 suppression), Tanshinone IIA (miR-449a/ACSL4 regulation), Tongqiao Huoxue Decoction (reported ACSL4 ubiquitination); **(C)** Antioxidant defense (Nrf2/GPX4/GSH) — Gastrodin, Salidroside, Puerarin (Nrf2/HO-1 activation), Salvianolic acid B, Astragaloside IV (SLC7A11/GPX4 upregulation), Ginsenoside Rg1 (PI3K/Akt/Nrf2 activation). TCM formulas (Buyang Huanwu Decoction, Naotai Fang, Tongqiao Huoxue Decoction) are highlighted for their multi-target characteristics. Electroacupuncture (GV20-based acupoint combinations) is shown targeting the Nrf2/SLC7A11/GPX4 axis, based on evidence from cerebral ischemia models (101, 102). Most mechanisms shown are derived from cerebral ischemia or other non-VCI models (indirect evidence; see Table 1). Single compounds are depicted as green circles; formulas as green rectangles; molecular targets as orange nodes; signaling pathways as blue arrows.

### 4.2 TCM monomers/single compounds targeting ferroptosis

Before reviewing individual agents, two caveats should be stated. First, most of the evidence summarized below was obtained in cerebral ischemia–reperfusion or other non-VCI models (evidence tiers C and D); direct evidence from VCI models (tier B) is currently available only for a minority of agents, as indicated for each compound and in Table 1. Second, reductions in ROS, MDA, or iron levels alone do not establish ferroptosis-specific inhibition (see Section 2.6); unless ferroptosis-specific rescue or genetic experiments were performed, such effects are described here as ferroptosis-associated.

#### 4.2.1 Baicalein

Baicalein, a bioactive flavonoid isolated from Scutellaria baicalensis, has demonstrated potent anti-ferroptotic activity in multiple neurological disease models. Mechanistically, baicalein directly inhibits 12/15-lipoxygenase (12/15-LOX) activity, reduces lipid peroxidation, and chelates intracellular iron (83). In cerebral ischemia–reperfusion models (indirect evidence for VCI), baicalein treatment significantly preserved GPX4 expression, reduced MDA and iron levels, and ameliorated neuronal ferroptosis and cognitive impairment (84). Baicalein was originally identified as a potent natural ferroptosis inhibitor in a natural product library screen (83).

#### 4.2.2 Salvianolic acid B

Salvianolic acid B (Sal B), the most abundant water-soluble compound in *Salvia miltiorrhiza* (Danshen), exerts neuroprotective effects in cerebral ischemia–reperfusion injury, including inhibition of TLR4/MyD88-mediated inflammation (85) and attenuation of oxidative stress (86). Although the antioxidant and iron-modulating activities of Sal B suggest ferroptosis-modulating potential, these findings derive from ischemia–reperfusion rather than VCI models (indirect evidence), and ferroptosis-specific validation in VCI models is warranted.

#### 4.2.3 Gastrodin

Gastrodin, the primary bioactive component of Gastrodia elata, has shown neuroprotective effects against ferroptosis in cerebrovascular disease models. Gastrodin has been shown to inhibit ferroptosis after ischemic stroke by modulating the xCT/GPX4 and ACSL4/LPCAT3 pathways (87). Importantly, gastrodin is among the few TCM compounds with direct evidence in a VCI model: in bilateral common carotid artery occlusion (BCCAO) rats, gastrodin reduced hippocampal Fe^2+^ and MDA, restored GSH, upregulated the Nrf2/Keap1-GPX4 axis, and improved learning and memory, with ferrostatin-1 producing comparable benefits (68).

#### 4.2.4 Astragaloside IV

Astragaloside IV (AS-IV), the principal active compound of Astragalus membranaceus, has demonstrated anti-ferroptotic effects in cerebral ischemia–reperfusion injury through activation of the P62/Keap1/Nrf2 pathway, with consequent upregulation of SLC7A11 and GPX4 (88). AS-IV also protects neuronal mitochondria from oxidative stress-induced dysfunction (89). These findings, obtained in ischemia–reperfusion and *in vitro* models, constitute indirect evidence for VCI.

#### 4.2.5 Tanshinone IIA

Tanshinone IIA, a lipophilic compound from *Salvia miltiorrhiza*, inhibits neuronal ferroptosis and ameliorates cerebral ischemia–reperfusion injury by upregulating miR-449a, which suppresses ACSL4-mediated lipid peroxidation (90). This evidence derives from an ischemic stroke model and is therefore indirect for VCI.

#### 4.2.6 Salidroside

Salidroside, derived from *Rhodiola rosea*, reduces inflammation and brain injury after permanent middle cerebral artery occlusion in rats (91). Its anti-ferroptotic activity has so far been demonstrated in a renal ischemia–reperfusion model via PI3K/AKT signaling (92); evidence in neurological or VCI models is still lacking (exploratory evidence).

#### 4.2.7 Curcumin

Curcumin, the principal curcuminoid of turmeric (*Curcuma longa*), attenuates microglia-mediated neuroinflammation and neuronal ferroptosis after experimental subarachnoid hemorrhage, in association with Nrf2/HO-1/GPX4 activation (93); iron-chelating and radical-scavenging activities have additionally been documented in a rhabdomyolysis-associated renal injury model (94). Direct evidence in VCI models is currently lacking (indirect evidence).

#### 4.2.8 Other notable TCM monomers

Additional TCM-derived compounds with reported anti-ferroptotic activities, mostly in non-VCI or non-neurological disease models (indirect or exploratory evidence for VCI), include:

*Resveratrol*: Activates the SIRT1/Nrf2 pathway and attenuates ferroptosis in myocardial ischemia–reperfusion injury (non-neurological model) (95).
*Puerarin*: Modulates Nrf2/GPX4 signaling and protects HT-22 neuronal cells against glutamate-induced ferroptosis *in vitro* (82).
*Icariin*: Upregulates GPX4 and FTH1 and inhibits NCOA4-mediated ferritinophagy in a glucocorticoid-induced bone deterioration model (non-neurological model) (96).
*Ginsenoside Rg1*: Activates the PI3K/Akt/Nrf2 pathway and inhibits ferroptosis in sepsis-induced acute kidney injury (non-neurological model) (97).

### 4.3 TCM formulas targeting ferroptosis

It is important to distinguish isolated active compounds from multi-herb formulas. A mechanism established for a purified compound cannot be directly extrapolated to a complex formula, in which dozens of constituents may act additively, synergistically, or antagonistically, and in which composition varies with herb sourcing, processing, and preparation. Standardization and quality control are therefore essential when interpreting formula-level evidence.

#### 4.3.1 Buyang Huanwu Decoction

Buyang Huanwu Decoction (BYHWD), a classical TCM prescription for stroke recovery, has demonstrated significant anti-ferroptotic effects in cerebrovascular disease models (98). In an ischemic stroke model, BYHWD inhibited ferroptosis via activation of the Nrf2/GPX4 pathway (98); a meta-analysis of animal studies supports its efficacy in focal cerebral ischemia (99), and a small clinical study reported efficacy in acute ischemic stroke (100). This evidence derives from ischemic stroke rather than VCI models (indirect evidence).

#### 4.3.2 Naotai Fang

Naotai Fang, a clinical prescription for VCI, has been reported to improve cognitive function in VCI patients and animal models (17). Preclinical studies summarized in systematic reviews suggest that Naotai Fang may inhibit ferroptosis in hippocampal neurons through activation of the PI3K/Akt/Nrf2 pathway, enhancing GPX4 and SLC7A11 expression (18); these mechanistic details are currently supported mainly by secondary sources and require confirmation in original VCI-model studies.

#### 4.3.3 Tongqiao Huoxue Decoction

Tongqiao Huoxue Decoction (TQHXD), a blood-activating and stasis-resolving formula, has been reported to exert neuroprotective effects in cerebrovascular models, with proposed involvement of ferroptosis pathways (17, 18). Reported effects include reduced hippocampal iron deposition, upregulation of the GSH-GPX4 axis, and attenuation of lipid peroxidation (18); however, these mechanistic claims derive largely from secondary literature, and verification in original studies using VCI models is needed.

#### 4.3.4 Other TCM formulas

Danggui Shaoyao San: Modulates iron metabolism and inhibits neuronal ferroptosis (17).
Xuefu Zhuyu Decoction: Reduces oxidative stress and ferroptosis in cerebrovascular disease (18).

### 4.4 Acupuncture and ferroptosis regulation

Although acupuncture is a non-pharmacological modality distinct from herbal compounds and formulas, it is briefly discussed here because it is frequently combined with herbal medicine in the integrative management of VCI in China. Emerging evidence suggests that electroacupuncture may modulate ferroptosis-related pathways in cerebral ischemia models (indirect evidence for VCI). In middle cerebral artery occlusion (MCAO) rats, electroacupuncture at GV20 (Baihui), GV26 (Shuigou), SP6 (Sanyinjiao), and PC6 (Neiguan) reduced iron accumulation and MDA, restored GSH and GPX4, and attenuated ferroptosis (101); electroacupuncture at GV20, GV14 (Dazhui), LI11 (Quchi), and PC6 suppressed ferroptosis by promoting Nrf2 nuclear translocation and activating the Nrf2/SLC7A11/GPX4 pathway (102). The proposed anti-ferroptotic mechanisms of acupuncture include: (1) enhancement of cerebral blood flow and reduction of hypoperfusion-induced oxidative stress; (2) modulation of iron regulatory protein expression; and (3) activation of endogenous antioxidant defense systems (102). The experimental evidence for TCM interventions targeting ferroptosis is comprehensively summarized in Table 1.

## 5 Discussion and future perspectives

### 5.1 Current challenges and knowledge gaps

Despite the emerging evidence linking ferroptosis to VCI pathogenesis and the promising anti-ferroptotic effects of TCM, several critical challenges and knowledge gaps remain. First, the direct evidence for ferroptosis in human VCI brain tissue is still limited, with most studies relying on animal models and *in vitro* systems (9, 11). Considerable residual uncertainty remains: the number of studies performed in bona fide VCI models is small, much of the mechanistic narrative is extrapolated from ischemic stroke, traumatic brain injury, or neurodegenerative disease, and pharmacological or genetic evidence establishing ferroptosis as a causal driver, rather than a consequence, of VCI progression is scarce. Many of the mechanistic explanations presented in this review should therefore be regarded as biologically plausible hypotheses awaiting experimental validation in VCI-specific settings. The development of specific ferroptosis biomarkers for clinical diagnosis and monitoring of VCI remains an unmet need.

Second, the relative contribution of ferroptosis compared to other forms of cell death (apoptosis, necroptosis, pyroptosis) in VCI has not been systematically evaluated (53). Ferroptosis is therefore best regarded not as the primary driver of VCI but as one component of a broader pathological network in which multiple cell-death modalities, oxidative stress, and inflammation operate simultaneously and interact. Understanding their hierarchical relationships and temporal dynamics is essential for therapeutic targeting, and combinatorial interventions may ultimately prove more effective than ferroptosis inhibition alone.

Third, the clinical translation of TCM interventions faces substantial challenges. The pharmacokinetic limitations of many TCM compounds, including low oral bioavailability, poor BBB penetration, and rapid metabolism, make it uncertain whether experimentally effective concentrations are achievable in the human brain (103). Effective doses, exposure-response relationships, and long-term safety remain poorly characterized, and potential herb-drug interactions with standard VCI medications (antiplatelet agents, anticoagulants, antihypertensives) require systematic evaluation. For herbal formulas, standardization, quality control, and batch-to-batch variability are additional unresolved obstacles to reproducibility and regulatory approval. Advanced drug delivery systems, such as nanoparticle-based formulations and exosome-mediated delivery, may offer partial solutions to these challenges.

### 5.2 Emerging research directions

Several promising research directions merit further investigation:

Multi-omics approaches: Integration of transcriptomics, proteomics, metabolomics, and lipidomics data will provide a comprehensive understanding of ferroptosis in VCI and identify novel therapeutic targets and biomarkers (9, 10).
Single-cell analysis: Single-cell RNA sequencing and spatial transcriptomics can reveal cell type-specific ferroptosis susceptibility and heterogeneity in VCI, enabling precision therapeutic strategies (104).
Network pharmacology: Computational approaches integrating TCM compound databases with ferroptosis-related target networks can systematically predict and validate the anti-ferroptotic mechanisms of TCM formulas, accelerating the identification of active components and therapeutic targets (105).
Clinical translation: Well-designed clinical trials evaluating the efficacy of TCM interventions on ferroptosis biomarkers and cognitive outcomes in VCI patients are urgently needed (81). The integration of traditional diagnostic criteria with modern neuroimaging and molecular biomarkers will facilitate personalized TCM treatment strategies.
Gut-brain axis: The gut microbiota modulates iron absorption, systemic inflammation, and brain ferroptosis, and TCM formulas may exert anti-ferroptotic effects partly through gut microbiota regulation (106).

### 5.3 Toward integrated therapeutic strategies

The integration of TCM anti-ferroptotic interventions with conventional VCI management represents a promising therapeutic paradigm. The multi-target nature of TCM is particularly advantageous for addressing the complex pathological network of ferroptosis in VCI, as single-target approaches may be insufficient to achieve meaningful clinical outcomes (107, 108). Future therapeutic strategies should consider: (1) combination of TCM with conventional vascular risk factor management; (2) stage-specific interventions targeting different ferroptotic mechanisms at different VCI stages; and (3) personalized treatment based on individual ferroptosis vulnerability profiles.

## 6 Conclusion

Ferroptosis has emerged as a potential pathological mechanism in VCI, with preclinical evidence implicating it in neuronal and glial cell injury through iron overload, lipid peroxidation, BBB disruption, white matter injury, neuroinflammation, and synaptic dysfunction. It should be noted, however, that direct evidence in VCI patients remains limited and that much of the supporting data derive from related cerebrovascular or non-neurological models. The multi-target, multi-pathway therapeutic approach of TCM provides a valuable arsenal of anti-ferroptotic agents, including both single compounds (baicalein, salvianolic acid B, gastrodin, astragaloside IV, tanshinone IIA) and classical formulas (Buyang Huanwu Decoction, Naotai Fang, Tongqiao Huoxue Decoction). These TCM interventions modulate ferroptosis through diverse mechanisms, primarily targeting the Nrf2/GPX4 axis, iron metabolism, and lipid peroxidation pathways.

However, the field remains in its early stages, with significant challenges in clinical translation, biomarker development, and mechanistic elucidation. Future research should prioritize: (1) validation of ferroptosis as a therapeutic target in human VCI through clinical studies; (2) development of specific ferroptosis biomarkers for VCI diagnosis and monitoring; (3) optimization of TCM formulations for enhanced bioavailability and BBB penetration; and (4) rigorous clinical trials evaluating the efficacy and safety of TCM anti-ferroptotic interventions in VCI patients.

By bridging the ancient wisdom of TCM with the modern understanding of ferroptosis biology, this integrative approach holds substantial promise for developing novel, effective therapeutic strategies for VCI, a devastating condition with limited current treatment options.
